# Sustainability in Surgery: Problems, Solutions, and Recommendations

**DOI:** 10.7759/cureus.94010

**Published:** 2025-10-07

**Authors:** Ayan Bin Rafaih, Kaso Ari

**Affiliations:** 1 Education, Aitchison College, Lahore, PAK; 2 Surgery, Norfolk and Norwich University Hospital, Norwich, GBR

**Keywords:** circular economy in healthcare, environmental impact of surgery, green operating rooms, healthcare waste management, surgical sustainability, sustainable healthcare

## Abstract

The healthcare sector contributes significantly to global carbon emissions, with surgical care representing a substantial portion of this environmental impact. This review examines the environmental challenges associated with modern surgical practice, exploring issues related to operating room waste, energy consumption, pharmaceutical waste, and the use of single-use devices. We analyse current solutions being implemented across healthcare systems, including waste reduction strategies, energy-efficient practices, sustainable procurement, and circular economy approaches. Finally, evidence-based recommendations are provided for healthcare administrators, surgical teams, and policymakers to create more environmentally sustainable surgical practices while maintaining high standards of patient care. This article emphasizes that sustainable surgical practice is not only an environmental imperative but also presents opportunities for cost savings and improved healthcare delivery.

## Editorial

Healthcare delivery systems account for approximately 4.4% of global greenhouse gas emissions, with a carbon footprint equivalent to the airline industry [1]. Within healthcare, surgical services are particularly resource-intensive, with operating rooms (ORs) generating 20-33% of hospital waste despite occupying a relatively small hospital footprint [2]. Addressing sustainability in surgery has become an urgent concern as healthcare systems face increasing pressure to reduce their environmental impact while maintaining or improving the quality of care and managing costs.

The concept of the "triple bottom line" in healthcare sustainability encompasses three interconnected domains: environmental stewardship, social responsibility, and economic prosperity [3]. Surgical sustainability initiatives must therefore balance ecological considerations, patient outcomes, and fiscal constraints. This integrated approach recognizes that sustainable practices can simultaneously reduce environmental harm, improve public health, enhance surgical quality, and generate economic benefits.

This article examines the environmental challenges associated with modern surgical practice, explores emerging solutions, and provides evidence-based recommendations for creating more sustainable surgical systems. We systematically analyse the environmental impact of operating rooms (including waste from single-use drapes and energy-intensive ventilation systems), surgical supply chains, and perioperative care pathways, while highlighting opportunities for improvement across these domains.

Environmental impact of modern surgical practice

Waste Generation in Operating Rooms

Operating rooms are significant contributors to hospital waste streams. A single surgical procedure can generate between 2 and 14 kg of waste, with complex surgeries producing substantially more [4]. Surgical waste encompasses several distinct categories. A large proportion consists of disposable surgical instruments and supplies, including single-use devices, drapes, gowns, gloves, and packaging materials. Another important category is biohazardous waste, which refers to materials contaminated with blood or other bodily fluids. Pharmaceutical waste represents a further component and typically includes unused medications as well as residual anaesthetic gases. Finally, operating rooms generate general waste such as paper, cardboard, and other non-contaminated materials.

The majority of OR waste is routinely classified as regulated medical waste requiring specialized disposal methods such as incineration or autoclaving, despite studies suggesting that up to 85% could potentially be classified as general waste [5]. This misclassification increases both financial and environmental costs of waste disposal.

Energy Consumption

Operating rooms are among the most energy-intensive spaces in hospitals, using between three and six times more energy per square foot than other clinical areas [6]. This heightened demand arises from several factors. Strict heating, ventilation, and air conditioning (HVAC) systems are required to maintain precise ventilation and temperature control, while medical equipment and patient monitoring systems often run continuously. Bright, high-intensity lighting further increases energy consumption, and sterilization processes place an additional load on resources. Additionally, many operating rooms operate 24/7 with minimal energy conservation measures during non-operational periods, further increasing energy consumption [6].

Anaesthetic Gases and Climate Impact

Inhaled anesthetic agents, particularly desflurane, sevoflurane, and isoflurane, are potent greenhouse gases with global warming potentials significantly higher than CO₂. Anaesthetic gases account for approximately 5% of the carbon footprint of healthcare institutions [7]. Most anaesthetic gases are vented directly into the atmosphere without capture or breakdown, contributing to climate change.

Single-Use Devices and Resource Consumption

The shift from reusable to single-use devices (SUDs) has been driven by concerns about infection control, convenience, and in some cases, economic factors. However, this transition has significantly increased resource consumption and waste generation. Life cycle assessments comparing single-use versus reusable surgical instruments consistently demonstrate higher environmental impacts associated with disposables across multiple environmental indicators [8].

Single-use medical devices carry a significant environmental burden. Their production requires the extraction of additional raw materials and consumes a considerable amount of energy during manufacturing. Once produced, these items also generate greater emissions through transportation, only to end their life cycle as medical waste, adding to the demands of disposal systems.

Water Usage in Surgical Settings

Surgical departments are also among the heaviest users of water within healthcare facilities. Large volumes are required for cleaning and sterilizing surgical instruments, as well as for routine scrubbing and handwashing procedures. Water is further consumed in equipment cooling systems and in the daily cleaning and maintenance necessary to keep these environments safe and sterile. A single surgical case can require 100-300 litres of water when accounting for all perioperative processes [9]. Water conservation in surgery represents an often-overlooked opportunity for sustainability improvements.

Current solutions and innovative approaches

Waste Reduction and Management Strategies

Proper waste segregation is fundamental to reducing the environmental impact of surgical waste. Implementing clear waste segregation protocols can significantly reduce the volume of waste that requires high-energy disposal methods. Studies have demonstrated that comprehensive recycling programs in operating rooms can divert 20-30% of waste from landfills or incineration [10].

A few recycling initiatives have proven effective in surgical settings. Blue wrap, the polypropylene material used to maintain sterility, can be collected and repurposed into new plastic products. Clear plastic packaging from surgical supplies is often recyclable through conventional streams, while many hospitals are transitioning from single-use blue wrap to reusable rigid containers for sterilization. Additionally, paper and cardboard packaging materials can be collected and recycled, thereby reducing the overall waste footprint of surgical waste.

Custom procedure trays (CPTs) provide pre-packaged sets of instruments and supplies for specific surgical procedures. While designed for convenience, poorly optimized CPTs can lead to significant waste when unused items are discarded. Several hospitals have introduced CPT review programs, which bring together multidisciplinary teams to optimize surgical supply use. These teams work to identify and eliminate items that are consistently unused, standardize instrument trays across similar procedures, and assess the necessity of single-use components. Such efforts not only reduce waste but also promote more efficient and cost-effective surgical practices. These optimization efforts have reduced waste by 20-30% in some institutions while generating significant cost savings [11].

The FDA-regulated reprocessing of certain single-use devices offers a way to reduce medical waste without compromising patient safety. Through this process, third-party reprocessors collect, clean, test, sterilize, and repackage eligible devices so they can be safely used multiple times. Commonly reprocessed items include external fixation components, select laparoscopic instruments, sequential compression sleeves, and electrophysiology catheters. Reprocessing programs have demonstrated waste reduction of 5,000-15,000 kg annually per institution with cost savings of 40-50% compared to new device purchases [12].

Energy Conservation and Efficiency

Modern sustainable operating room design integrates a range of energy-efficient features. LED surgical lighting can reduce energy use by 50 to 80 percent compared with traditional halogen systems while producing less heat. Smart HVAC systems adjust air exchange rates according to occupancy and surgical activity, and occupancy sensors further reduce energy consumption by dimming lights and limiting ventilation when rooms are unoccupied. In addition, heat recovery systems capture waste heat from ventilation and redirect it for use elsewhere in the facility, further enhancing overall efficiency.

Anaesthetic Gas Management

Several strategies can help reduce the climate impact of anaesthetic gases. Low-flow anaesthesia minimizes fresh gas flow during the maintenance phase, while total intravenous anaesthesia (TIVA) replaces inhaled agents with intravenous alternatives when clinically appropriate. Selecting anaesthetics with lower global warming potential further mitigates environmental effects, and emerging capture and reclamation technologies offer the potential to collect exhaled anaesthetic gases for safe reuse or disposal. Implementation of anaesthetic gas reduction programs has demonstrated potential carbon emission reductions equivalent to removing 200-300 cars from the road per institution annually [13].

Sustainable procurement and supply chain

Environmentally Preferable Purchasing

Environmentally preferable purchasing (EPP) programs enable hospitals to use their purchasing power to favour products with lower environmental impacts. In surgical settings, effective EPP programs incorporate standardized environmental criteria into procurement decisions, utilize supplier sustainability scorecards and metrics, and consider lifecycle costs rather than focusing solely on acquisition price. Hospitals also collaborate with suppliers to reduce packaging waste, further minimizing the environmental footprint of surgical operations. Successful EPP initiatives have reduced packaging waste by 15-25% while maintaining or reducing costs [14].

Local and Regional Sourcing

Shortening supply chains through local and regional sourcing can markedly reduce transportation-related emissions while enhancing supply chain resilience. This approach decreases delivery distances and associated emissions, speeds up delivery times, allows for leaner inventories, improves flexibility and responsiveness, and supports local economies. While not all surgical supplies can be sourced locally, targeted regionalization of appropriate categories can reduce transportation-related emissions by 30-50% for those items [15].

Circular economy approaches in surgery

Instrument Reprocessing and Refurbishment

Extending the lifespan of surgical instruments through careful maintenance, repair, and refurbishment is a key strategy within the circular economy. Hospitals implement programs that ensure regular maintenance and refurbishment of reusable instruments, prioritize repair over replacement whenever possible, and recover materials from instruments that have reached the end of their usable life. These programs can extend instrument lifespan by 3-5 years while reducing procurement costs and waste generation [16].

Materials Recovery and Repurposing

Innovative strategies for recovering materials from surgical waste streams are increasingly being adopted [17]. Sterilization wrap, for example, can be repurposed into products such as tote bags or construction materials, while PVC from IV bags and tubing can be collected and recycled for new uses. Hospitals are also exploring methods to extract valuable metals from surgical instruments and equipment, further reducing waste and conserving resources.

Barriers to implementation

Regulatory and Compliance Challenges

Stringent infection control regulations can sometimes clash with sustainability objectives, especially when it comes to reusing medical devices. In some jurisdictions, reprocessing of single-use devices is restricted, and the regulatory pathways for introducing innovative sustainable technologies can be complex [18,19]. Differing international standards create additional compliance challenges, while risk-averse institutional policies often favour disposable items over reusable alternatives.

Economic Considerations

Financial barriers can also hinder the adoption of sustainable practices in surgical settings. Upfront capital costs for energy-efficient equipment and infrastructure can be prohibitive, and split incentives arise when departments managing waste budgets are separate from those purchasing supplies. Procurement decisions often overlook life-cycle costs, and reimbursement models rarely account for sustainability initiatives, making it more difficult for hospitals to invest in environmentally responsible practices.

Knowledge and Awareness Gaps

Awareness of the environmental impact of surgical practice remains limited among many healthcare professionals. Surveys suggest that 60 to 70 percent of surgical staff underestimate the energy and resource demands of operating rooms, while only 20 to 30 percent have received formal training on sustainability practices [20-22]. Additionally, 40 to 50 percent of staff report uncertainty about correct waste segregation procedures, highlighting the need for targeted education and training initiatives.

Operational and Logistical Challenges

Implementing sustainable practices in surgical settings also faces practical challenges. Limited space can make waste segregation and recycling difficult, while time pressures often restrict the adoption of new practices. Transitioning to updated systems may disrupt established workflows, and staff resistance to changing long-standing routines can further complicate efforts.

Recommendations for sustainable surgical practice

For Healthcare Administrators and Institutions

Effective governance and leadership are critical for advancing sustainability in surgical settings. Hospitals can establish formal sustainability committees that include representatives from surgical departments, integrate environmental metrics into institutional performance dashboards, and develop clear policies and protocols tailored to the specific needs of surgical services. Allocating resources to sustainability initiatives with clearly defined expectations for return on investment further supports successful implementation [23].

Infrastructure and systems play a critical role in promoting sustainability in surgical settings. Investing in energy-efficient operating room technologies and building systems helps reduce resource consumption, while comprehensive waste segregation infrastructure ensures proper recycling and disposal. Hospitals can also implement data collection systems to track key environmental performance indicators, providing insight into areas for improvement. Moreover, considering sustainability in facility design and renovation projects ensures that both new and updated spaces support long-term environmental goals.

Sustainable procurement and supply chain practices are essential for reducing the environmental footprint of surgical services. Hospitals can implement environmentally preferable purchasing policies for surgical supplies and work with suppliers to reduce packaging waste. Evaluating reusable alternatives for high-volume disposable items further minimizes resource use, while considering the total cost of ownership rather than focusing solely on acquisition cost supports more sustainable, long-term decision-making.

For Surgical Teams and Clinicians

Modifying clinical practices can play a significant role in enhancing sustainability in surgical settings. Low-flow anaesthesia techniques should be adopted when clinically appropriate, and regional or total intravenous anaesthesia can be considered based on patient needs. Clinicians are encouraged to critically evaluate the necessity of supplies before opening packaging and to prioritize reusable instruments and equipment when they are clinically equivalent to disposable alternatives.

Education and awareness are vital for fostering sustainable practices in surgical settings whilst maintaining safety [24]. Sustainability topics should be incorporated into surgical training programs, and regular in-service sessions can reinforce proper waste segregation protocols. Sharing data on departmental environmental performance helps staff understand the impact of their actions, while recognizing and celebrating sustainability champions within surgical teams encourages ongoing engagement and leadership in environmentally responsible practices.

Innovation and research are key drivers of sustainability in surgical care. Hospitals and surgical teams can participate in evaluating new sustainable technologies and practices, while documenting and publishing the outcomes of these initiatives helps share best practices. Collaboration with industry partners supports the development of environmentally friendly surgical products, and advocating for the inclusion of environmental metrics in surgical quality frameworks ensures that sustainability becomes an integral part of healthcare performance evaluation.

For Policymakers and Professional Organizations

A robust regulatory framework is essential for advancing sustainability in surgical settings. Clear guidelines should be developed for the safe reprocessing of appropriate single-use devices, while efforts to harmonize international standards for medical waste classification can simplify compliance. Creating incentives for healthcare facilities to invest in sustainability infrastructure encourages the adoption of environmentally responsible practices, and incorporating environmental criteria into healthcare facility accreditation standards ensures that sustainability is recognized as a core component of quality care.

Cultivating a culture of sustainability in surgical practice begins with professional education. Sustainability should be integrated into core competencies for surgical training, and specialty-specific guidelines can provide practical direction for environmentally responsible care. Including environmental topics in continuing medical education ensures ongoing awareness, while recognition programs for sustainability leadership highlight and reward clinicians who champion sustainable practices within their teams.

Implementation Framework and Timeline

Implementing comprehensive sustainability programs in surgical settings requires a structured, stepwise approach that can be adapted with strategies already tested [25-30]. Phase 1, the assessment and baseline phase, typically lasts three to six months and involves conducting waste audits to identify opportunities, establishing environmental performance metrics, forming a multidisciplinary sustainability committee, and targeting high-impact initiatives that are relatively easy to implement. Phase 2, the early implementation phase, spans six to twelve months and focuses on deploying waste segregation infrastructure, launching staff education programs, piloting sustainable practices in selected operating rooms, and beginning data collection on key metrics. Phase 3, the expansion and integration phase, occurs over one to two years, during which successful initiatives are extended across all surgical areas, sustainability criteria are incorporated into procurement processes, energy conservation measures are implemented, and recognition programs are developed to celebrate environmental achievements. Finally, Phase 4, the continuous improvement phase, is ongoing and emphasizes regular review and optimization of initiatives, benchmarking performance against other institutions, fostering innovation and research partnerships, and publicly reporting environmental outcomes to promote accountability and continuous advancement.

Conclusion

Sustainability in surgery represents both a challenge and an opportunity for healthcare systems worldwide. The environmental impact of surgical practice is substantial, but evidence demonstrates that significant improvements are achievable through systematic implementation of waste reduction, energy conservation, circular economy approaches, and sustainable procurement. Moving toward sustainable surgical practice requires multifaceted interventions across clinical, operational, and policy domains. Success depends on engagement from healthcare administrators, clinical teams, industry partners, and regulatory bodies working in concert toward common goals.

While barriers to implementation exist, the potential benefits extend beyond environmental impact to include cost savings, improved operational efficiency, and enhanced quality of care. Sustainability in surgery is not merely an environmental imperative but a pathway to more resilient, effective, and responsible healthcare delivery. Future research should focus on quantifying the clinical, economic, and environmental outcomes of sustainability initiatives to build the evidence base for wider adoption. Standardized metrics and reporting frameworks will facilitate benchmarking and identification of best practices across institutions and healthcare systems. By embracing sustainability principles, the surgical community has the opportunity to significantly reduce its environmental footprint while maintaining excellence in patient care, demonstrating that environmental stewardship and clinical quality are complementary rather than competing priorities.
